# Coordinate Fault Ride-Through Strategy for Connection of Offshore Wind Farms Using Voltage Source-Converter-Based High-Voltage Direct-Current Transmission under Single Polar Fault

**DOI:** 10.3390/s23125760

**Published:** 2023-06-20

**Authors:** Huiying Zhou, Siyang Ge, Liang Qin

**Affiliations:** School of Electricity and Automation, Wuhan University, No. 299, Bayi Road, Luojiashan Street, Wuchang District, Wuhan 430072, China; z15375095473@163.com (H.Z.); gesiyang123@163.com (S.G.)

**Keywords:** VSC-HVDC, DFIG, DC overcurrent, crowbar, coordinated FRT strategy

## Abstract

In a system where wind farms are connected to the grid via a bipolar flexible DC transmission, the occurrence of a short-time fault at one of the poles results in the active power emitted by the wind farm being transmitted through the non-faulty pole. This condition leads to an overcurrent in the DC system, thereby causing the wind turbine to disconnect from the grid. Addressing this issue, this paper presents a novel coordinated fault ride-through strategy for flexible DC transmission systems and wind farms, which eliminates the need for additional communication equipment. The proposed strategy leverages the power characteristics of the doubly fed induction generator (DFIG) under different terminal voltage conditions. By considering the safety constraints of both the wind turbine and the DC system, as well as optimizing the active power output during wind farm faults, the strategy establishes guidelines for the wind farm bus voltage and the crowbar switch signal. Moreover, it harnesses the power regulation capability of the DFIG rotor-side crowbar circuit to enable fault ride-through in the presence of single-pole short-time faults in the DC system. Simulation results demonstrate that the proposed coordinated control strategy effectively mitigates overcurrent in the non-faulty pole of flexible DC transmission during fault conditions.

## 1. Introduction

With the rapid development of power electronic-control technology, in the wind farm grid and transmission of a variety of feasible solutions, the use of a two-pole VSC-HVDC system grid-connected transmission solution has gradually become more widely used and promoted. However, as new opportunities appear, so do challenges [1].

Grid-connected systems for wind farms with flexible direct current transmission (VSC-HVDC) usually use a bipolar operation system with high reliability [2,3]. Compared to conventional pseudo-bipolar operation, when one pole of a bipolar system is out of service, the other pole can still operate normally and maintain half of its rated transmission capacity because the two poles are independent of each other [4].

This feature of bipolar operation systems aims to improve the reliability and stability of grid-connected operation, but for wind farms connected to the grid via a bipolar VSC-HVDC system, the wind farm side converter (WFVSC) of the VSC-HVDC needs to provide voltage and frequency support to the wind farm, so constant AC voltage control is generally used. When a fault such as DC-side grounding, disconnection, or valve-side grounding occurs on one pole of a two-pole system, the power-transfer capability of that pole will drop to 0 [5]. Since the WFVSC on the other pole can still maintain constant AC voltage control during the fault, the wind farm cannot sense the fault occurring in the DC system when the fault occurs. In a short period of time, without considering the wind-power change, the wind turbine basically keeps the active power output unchanged during a flexural fault. Without adding additional control, the non-faulted pole of the DC system generates a severe overcurrent, which leads to DC system lockout and the wind farm going off-grid, with impact on the grid.This is the most likely to fail problem during the operation of wind farms via a flexible DC transmission grid-connected system, and it is also a problem that must be solved for the development of a stable wind power grid-connected system.

The current control methods for the joint-fault ride-through of wind power and flex-direct include several types of methods including voltage reduction, frequency boosting, and communication methods [6]. The step-down method is a method to achieve rapid power reduction by significantly reducing the voltage at the end of the machine in a short period of time while maintaining the rated current [7,8]. Due to the rapid magnitude and rate of AC voltage reduction, strong shaft stress and rotor-side overcurrent will be generated inside the DFIG, while the drastic fluctuation of AC voltage will lead to system instability. By establishing a relationship between the frequency reference value of the wind farm side converter and the active power reference value of the wind turbine, the frequency step-up method reduces the active power reference value and thus reduces the active output of the wind turbine by increasing the wind farm outlet frequency during a DC system fault [9,10]. However, its application is somewhat limited by the problem of the slow response of the fan to sense frequency changes through the phase-locked loop. Both of these methods lead to an increase in fan rotor speed as well as an increase in fan DC bus voltage, so auxiliary controls are needed to suppress fan rotor speed as well as excessive DC bus voltage while fault traversal is taking place. Energy-consuming resistors occupy a large area in the field and are not economical. The application of the elevated frequency method is somewhat limited due to its slow response rate. The communication method sends the power reduction factor calculated by WFVSC to the wind farm to reset the active power reference value of the converter and thus reduce the EM power output [11]. The disadvantage of this method is that the communication delay limits the response speed of power control and requires high communication reliability. In summary, the existing fault ride-through methods have more or less insurmountable shortcomings, and with the further development of wind power grid connection, new requirements for fast and safe fault ride-through strategies and a better solution are needed.

In this study, a coordinated control strategy is proposed for a bipolar flexible DC transmission system and a wind farm, aiming to address the challenges associated with fault ride-through. The strategy leverages the power characteristics of a doubly fed wind turbine (DFIG) under different terminal voltage conditions to establish a relationship between the wind farm bus voltage and the crowbar switching signal. By utilizing the power regulation capability of the crowbar circuit inside the DFIG, the fault ride-through of the DC system is effectively achieved.

This method employs the end-of-machine voltage as the communication signal, eliminating the need for additional control loops and communication ports within the turbine. Through appropriate adjustments to the end-of-machine voltage and internal impedance, the power output of the wind turbine is suppressed, ensuring that the non-fault pole DC line does not create an overcurrent during a fault. Notably, this approach offers several advantages, including real-time responsiveness, no disruption to the working state of the wind turbine and DC system after fault resolution, no communication requirements throughout the fault ride-through process, and the ability to reduce active power during faults while maximizing the transmission capacity of the non-faulted pole. These advantages effectively address and optimize various issues associated with existing methods, thereby significantly improving the fault ride-through capability.

Overall, the proposed coordinated control strategy represents a significant advancement in fault ride-through techniques for wind farms. By effectively solving the inherent challenges and enhancing the fault ride-through process, this strategy contributes to the achievement of fault ride-through objectives and promotes the overall reliability and stability of wind farm integration with the grid.

## 2. Modeling of a Wind Farm Grid-Connected System Via Bipolar Flexible DC Transmission

**Figure 1:**
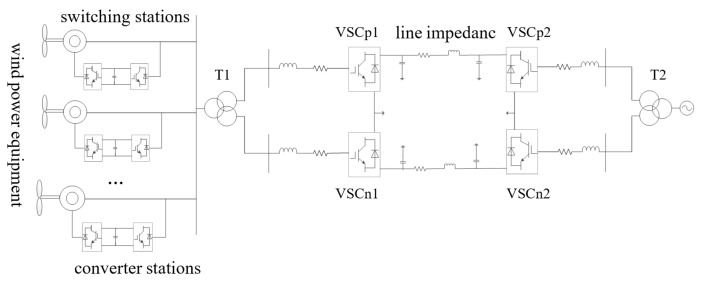
System outline of a VSC-HVDC system for wind farm connection.

The structure of a wind farm connected to the grid via a bipolar flexible DC transmission system is shown in Figure 1, with the wind farm and AC network on each side. The wind turbine is composed of a double-fed wind turbine generator (DFIG) commonly used today. T1 and T2 are the converter transformers on the wind farm side and the grid side, respectively; VSCp1, VSCp2, VSCn1, and VSCn2 are the mutually independent converter stations at each end.

The grid-side converter of the flexible DC system needs to provide DC voltage support for the stable operation of the DC system to ensure the system power balance, and thus uses constant DC voltage control [12], and the wind farm-side converter uses constant AC voltage control thus provides the necessary voltage and frequency support to the wind turbine. The control block diagram is shown in Figure S1a,b in Supplementary Materials.

The doubly fed wind turbine uses a back-to-back voltage-based PWM converter for AC excitation. The control goal of the rotor-side converter is to achieve variable speed and constant frequency operation and maximum wind energy tracking; the control goal of the grid-side converter is to keep the DC bus voltage constant and the power factor controllable. Therefore, the rotor-side converter uses constant active power control and constant reactive power control, and the network-side converter uses double closed-loop control of the DC link voltage and current [13,14,15], and the control block diagrams are shown in Figure S2a,b in Supplementary Materials.

## 3. Power Regulation Characteristics of DFIG after Crowbar Access

The crowbar circuit is a protection circuit installed inside the DFIG to protect the DFIG by quickly shorting the DFIG rotor during the stator voltage dip [16,17]. The type of crowbar protection circuit used in this paper is the IGBT-type crowbar circuit, as shown in Figure S3 in Supplementary Materials.

For the conventional fixed-resistance crowbar circuit, the DFIG cannot freely adjust the output electromagnetic power after the crowbar is connected. Since the wind farm grid-connected voltage of the combined system is controlled by WFVSC, which is different from the AC grid voltage. Therefore, it is possible to use the converter station’s control of the wind farm bus voltage, combined with the crowbar circuit, so that the crowbar circuit can adjust the absorbed EM power as needed during a fault, thus making the DFIG’s output active power controllable. With this control strategy, the purpose of fault ride-through can be achieved while maximizing the transmission capacity of the non-faulted pole by reducing the active power generated during the fault.

When the crowbar circuit is connected, the DFIG can be treated as a wirewound asynchronous motor with series resistance [18,19]. During the analysis of the motor, the following assumptions are made: (1) to ignore the stator–rotor resistanceand the stator transient process; (2) ignore the change in the differential rotation before and after putting in the crowbar; (3) ignore the effect of the network-side converter current on the stator current because the current provided by the network-side converter is very small [20]. According to the above assumptions, the expression of electromagnetic power of DFIG at this time is shown in the following equation, and the specific derivation is shown in Supplementary Materials:

(1) $$P_{em}=f\left(Z,\omega_{s}\right)\cdot u_{s}^{2}$$

where $u_{s}$ is the stator voltage and $f(Z,\omega_{s})$ is a function of generator impedance and rotational speed, depending on the generator’s internal impedance Z and the rotational speed $\omega_{s}$ [21].

The wind power input to the wind turbine can be expressed as:

(2) $$P_{w}=\frac{1}{2}\rho S_{w}\nu_{w}^{3}$$

where ρ is the air density, $S_{w}$ is the blade windward sweeping area, and $\nu_{w}$ is the wind speed.

The relationship between the motor speed and wind speed of DFIG satisfies the equation:

(3) $$\omega_{s}=\frac{\lambda_{opt}\nu_{w}}{R_{w}}$$

where $\lambda_{opt}$ is the optimal blade-tip speed ratio and $R_{w}$ is the wind turbine radius.

According to the correspondence between wind speed and motor speed, shown in (2) and (3), when the wind power $P_{w}$ injected into the wind turbine changes, the rotor speed will also change accordingly. In this paper, the wind power is used to characterize the rotor speed at different operating points.

**Figure 2:**
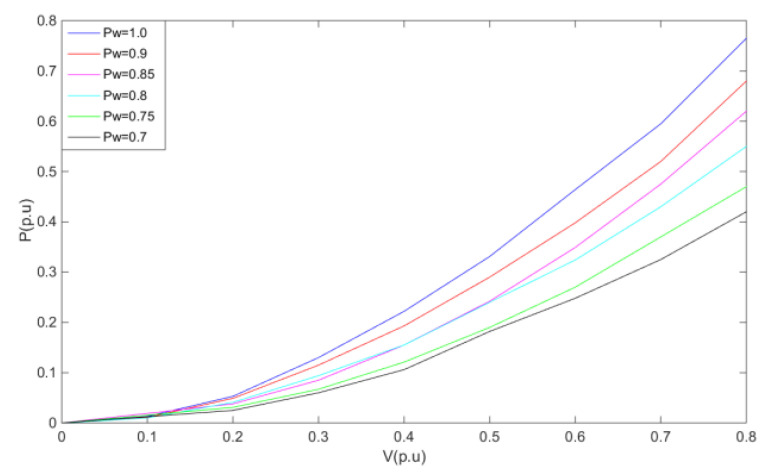
The fitting curve of active power and terminal voltage.

In the analysis of the transient process, the following assumption is made to simplify the study of the problem, i.e., due to the short DC line fault time and the large mechanical inertia of the DFIG system, it can be assumed that the rotational speed of the generator is basically constant during the whole DC line fault [22]. Based on the above assumption, the output active power versus the machine terminal voltage curve of DFIG under different wind power is plotted according to (1), as shown in Figure 2. From (1) and Figure 2, it can be seen that the output active power of DFIG is approximated as a quadratic function of the stator voltage $u_{s}$ when the wind power is determined and the crowbar circuit is switched to the full-on state.

The equivalent current through the crowbar resistor is shown in (4):

(4) $$I_{r}=D\frac{U_{d}}{R_{cb}}$$

where D is the duty cycle of the crowbar circuit IGBT, $R_{eq}$ is the resistance of the energy drain resistor, and $U_{d}$ is the crowbar DC side voltage.

The equivalent resistance $R_{eq}$ of the crowbar circuit is shown in (5):

(5) $$R_{eq}=\frac{U_{d}}{I_{r}}=\frac{R_{cb}}{D}$$

By adjusting the duty cycle D, the equivalent resistance $R_{eq}$ of the crowbar circuit can be adjusted. According to (1), the expression of the output electromagnetic power of the wind turbine during a fault is related to the internal impedance of the DFIG, and adjusting the duty cycle can change the output power of the wind turbine.

The expression for the electromagnetic power output from the DFIG during the crowbar action is derived in Supplementary Materials as shown in (6).

(6) $$P_{e}=\frac{s\omega_{s}^{2}\sigma L_{m}L_{s}L_{r}\left[L_{r}^{2}R_{s}+L_{m}^{2}R_{r}+\left(s^{2}\omega_{s}^{2}\right)\cdot \left(L_{r}^{2}R_{s}+L_{s}L_{r}R_{r}\right)\right]u_{sd}^{2}}{{\left(R_{r}L_{s}+R_{s}L_{r}\right)}^{2}+{\left(\omega_{s}\sigma L_{r}L_{s}\right)}^{2}}$$

where, s is the rotation rate; $u_{sd}$ is the d-axis component of the stator phase voltage; $L_{m}$ is the mutual inductance between the stator and rotor coaxial equivalent windings in the dq coordinate system; $L_{s}$ and $R_{s}$ are the stator equivalent two-phase winding self-inductance and equivalent resistance in the dq coordinate system; $L_{r}$ and $R_{r}$ are the rotor equivalent two-phase winding self-inductance and equivalent resistance in the dq coordinate system; ω is the synchronous speed of DFIG.

Substituting (5) into (6) yields

(7) $$P_{e}=\frac{s\omega_{s}^{2}\sigma L_{m}L_{s}L_{r}\left[L_{r}^{2}R_{s}+L_{m}^{2}R_{cb}/D+\left(s^{2}\omega_{s}^{2}\right)\cdot \left(L_{r}^{2}R_{s}+L_{s}L_{r}R_{cb}/D\right)\right]u_{sd}^{2}}{{\left(R_{cb}L_{s}/D+R_{s}L_{r}\right)}^{2}+{\left(\omega_{s}\sigma L_{r}L_{s}\right)}^{2}}$$

**Figure 3:**
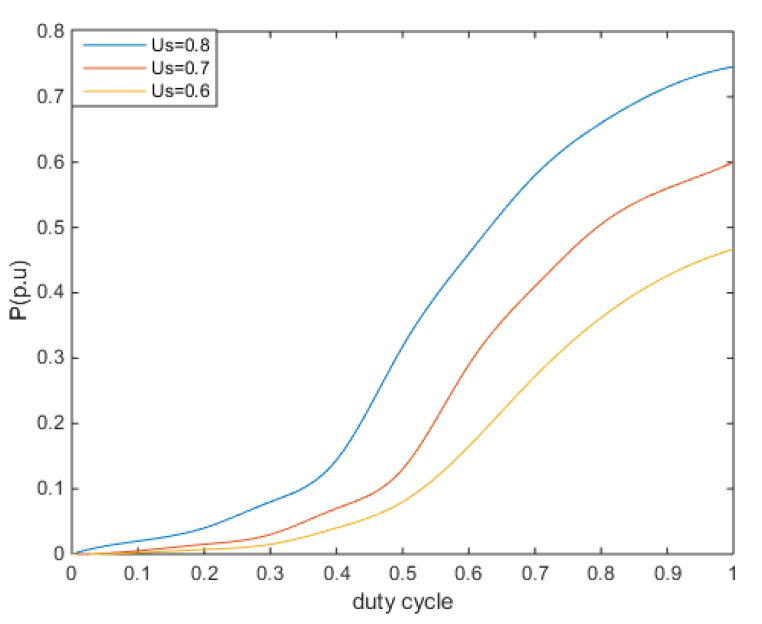
The fitting curve of electromagnetic power and duty cycle.

According to (7), the relationship curves between different machine terminal voltages and the switching duty cycle of crowbar and the output electromagnetic power of DFIG under rated wind power are made as shown in Figure 3.

In summary, after the crowbar circuit is put into operation, the electromagnetic power output from DFIG can be regulated by changing the machine terminal voltage and the equivalent resistance of the crowbar circuit. Based on the above principles this paper proposes a step-down coordinated fault ride-through strategy using the crowbar circuit.

## 4. Coordinated Fault Ride-Through Strategy

The conventional DFIG control is decoupled from the flexible DC transmission system, so the wind farm cannot sense faults in the DC system. The coordinated fault ride-through control described in this paper uses the wind farm bus voltage signal as a medium for information interaction between the two systems: When a single pole fault occurs in the DC system, the WFVSC issues a voltage-drop command, and when DFIG detects that the machine terminal voltage has dropped to a certain value, the crowbar circuit acts and stops the IGBT trigger-pulse signal of the rotor-side converter, at which point the coordinated fault ride-through control starts. When the fault is cleared and the machine terminal voltage returns to the rated value, the crowbar circuit exits and resumes the IGBT trigger-pulse signal of the rotor-side converter. During the entire fault ride-through process, the wind farm bus voltage-change signal acts as a communication command between the flexible DC system and the wind farm, with no communication requirements throughout the process. At the same time, the DFIG adjusts the output electromagnetic power in real time according to the change of the machine terminal voltage and the equivalent resistance of the crowbar circuit to achieve the purpose of fault ride-through.

The safety constraint of the non-faulted pole after a fault occurs should satisfy:

(8) $$I_{c}\leq \left(1-k\right)I_{cmax}$$

where $I_{c}$ is the converter current, $I_{cmax}$ is the current limit allowed to flow through the converter, and k is the current margin. In this paper, the current margin is set to 0.2.

According to (7), the electromagnetic power output of the DFIG during a fault can be changed by adjusting the wind farm AC bus voltage amplitude and the IGBT duty cycle of the DFIG rotor-side crowbar circuit to achieve fault ride-through. Therefore, the core of the coordinated fault ride-through control is to determine the voltage drop command from the WFVSC and the corresponding adjustment command of the IGBT duty cycle of the crowbar circuit. In this paper, the joint operation curve of WFVSC and the wind turbine during fault ride-through is designed according to the safety constraints of the DC system. During a single-pole fault in the DC system, the WFVSC changes the reference value of the AC bus voltage according to this operating curve, while the wind turbine adjusts the duty cycle of the crowbar circuit according to the change of the bus voltage and the input wind power, so that the power output of the wind turbine is suppressed by the change of the machine terminal voltage and the internal impedance, thus ensuring that the non-faulted DC line is never providing an overcurrent during the fault.

**Figure 4:**
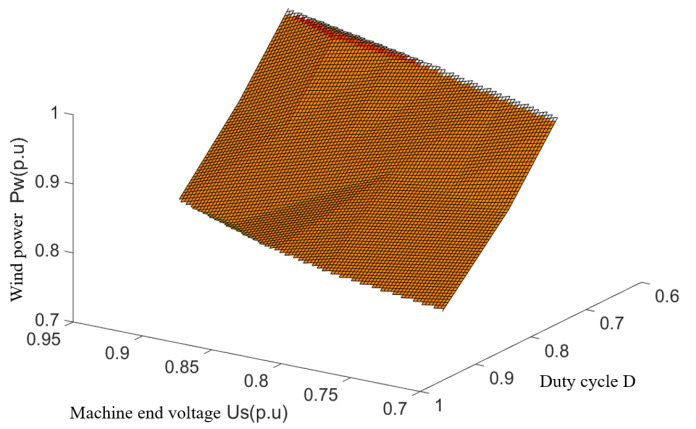
The boundary surface of security constraint.

Based on the current constraints shown in (8), the set of operating points ($P_{wn}$, $U_{sn}$, and $D_{n}$) in the fault ride-through when the current reaches the line limit can be determined from (7). A three-dimensional spatial fit to the above point set is used to draw a safety constrained boundary surface with wind power $P_{w}$, end voltage $u_{s}$; and crowbar circuit IGBT duty cycle D as variables. This is shown in Figure 4.

In the rectangular-space coordinate system shown in Figure 4, the electromagnetic power output of the wind turbine can be characterized by three quantities: wind power, machine terminal voltage, and crowbar circuit duty cycle. According to Figure 4, when the operating point of the DFIG is below the boundary surface, the output power of the DFIG satisfies the safety constraint requirement of the DC system, i.e., the non-fault pole converter station will not have overcurrent at this time.

**Figure 5:**
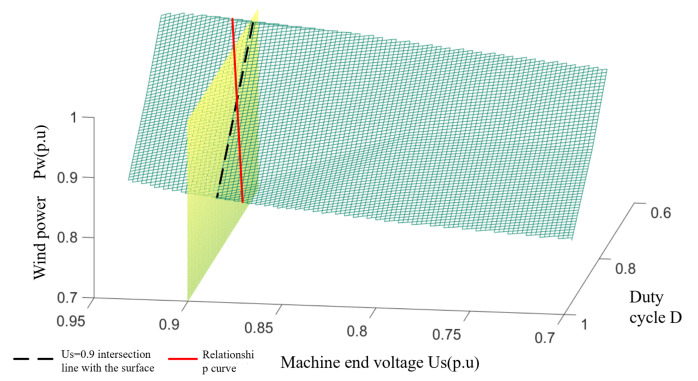
The relation curve of between terminal voltage, duty cycle, and wind power.

Since the same wind power on the surface corresponds to a combination of plural machine terminal voltages and crowbar duty cycle, the surface needs to be downscaled in order to construct a working curve usable in actual engineering. During the fault ride-through, the lower wind farm bus voltage will suppress the maximum active power that can be output by the wind turbine due to the limitation of the maximum current flowing through the WFVSC. Meanwhile, in order to make a one-to-one mapping relationship between the machine terminal voltage and crowbar duty cycle on the operating curve, the plane of $U_{s}=0.9\;p.u$ is rotated by a small angle along the $U_{s}$ − *D* plane in this paper, and its intersection with the relational surface is taken as the operating curve for the joint operation of WFVSC and wind turbine. This operating curve describes the relationship between the machine terminal voltage, duty cycle, and wind power in the fault ride-through strategy. The operating curve is shown in Figure 5.

When the wind power is lower than 50% of the rated value, the maximum value of current measured at the current measurement point of the non-faulted pole-converter station is still lower than the current limit of the trigger protection, at this time there is no need to use the fault ride-through strategy and the non-faulted pole will not overcurrent. Therefore, set the AC current threshold of WFVSC trigger coordination control $I_{th}=\left(1-k\right)I_{cmax}$ and start the fault ride-through strategy when the current $I_{c}$ of the converter station is greater than $I_{th}$ after the fault occurs.

**Figure 6:**
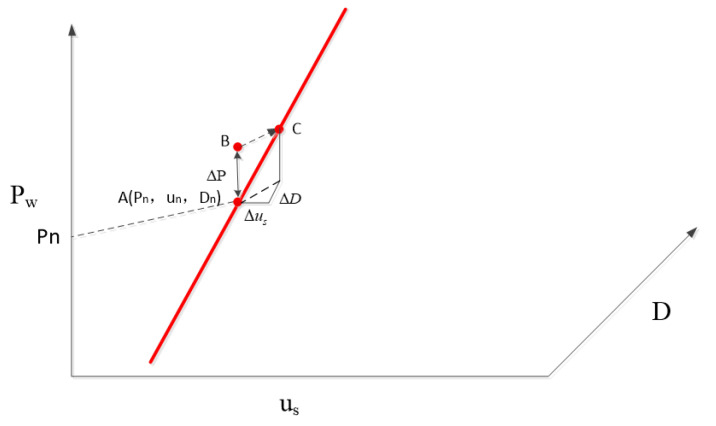
The movement trail of operating point during FRT.

**Figure 7:**
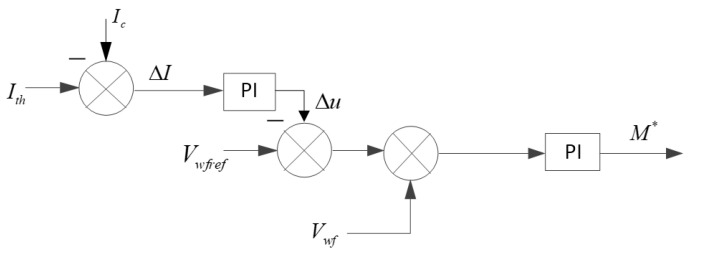
WFVSC voltage-control strategy.

Figure 6 gives the trajectory of the DFIG working state during the fault ride-through process. Point A ($P_{n}$, $u_{n}$, $D_{n}$) is the active output state of the DFIG when the wind power is $P_{n}$, the machine terminal voltage is $u_{n}$, and the duty cycle of the crowbar is $D_{n}$. As the wind power changes, the output active power of the wind turbine will also change, so the change in the rms value of the AC current at the measurement point of the converter station can be equated to the change in wind power. When the AC current $I_{c}$ measured by the converter station deviates from the reference value of current ΔI, the working state of DFIG at this time can be regarded as point B ($P_{n}+\Delta P$, $u_{n}$, $D_{n}$), point B is located outside the working curve, and the intersection of the plane $P_{w}=P+\Delta P$ and the operating curve C ($P_{n}+\Delta P$, u+Δu, D+ΔD) in the right angle coordinate system and the projection of BC on the $U_{s}-D$ plane can determine the machine terminal voltage regulation Δu and the duty cycle regulation ΔD of crowbar. Based on the above principle, the feedback control of the measured point current is established, $I_{th}$ is set as the current reference value of WFVSC, and the deviation of the AC current rms value is introduced into the voltage control of WFVSC. At the same time, a three-dimensional list with input as the stator voltage and wind power and output as the duty cycle is established in the wind farm control section, and the duty cycle corresponding to the machine terminal voltage and wind power can be obtained by off-line calculation according to the values of wind power and machine terminal voltage, and the IGBT of crowbar circuit is controlled.The voltage-control block diagram of WFVSC is shown in Figure 7.

Since this control method uses the machine terminal voltage as the communication signal, no additional control loops and communication ports need to be installed inside the wind turbine. The wind farm side converter can detect the non-fault pole current in real time, so this control method has a better real-time performance.

**Figure 8:**
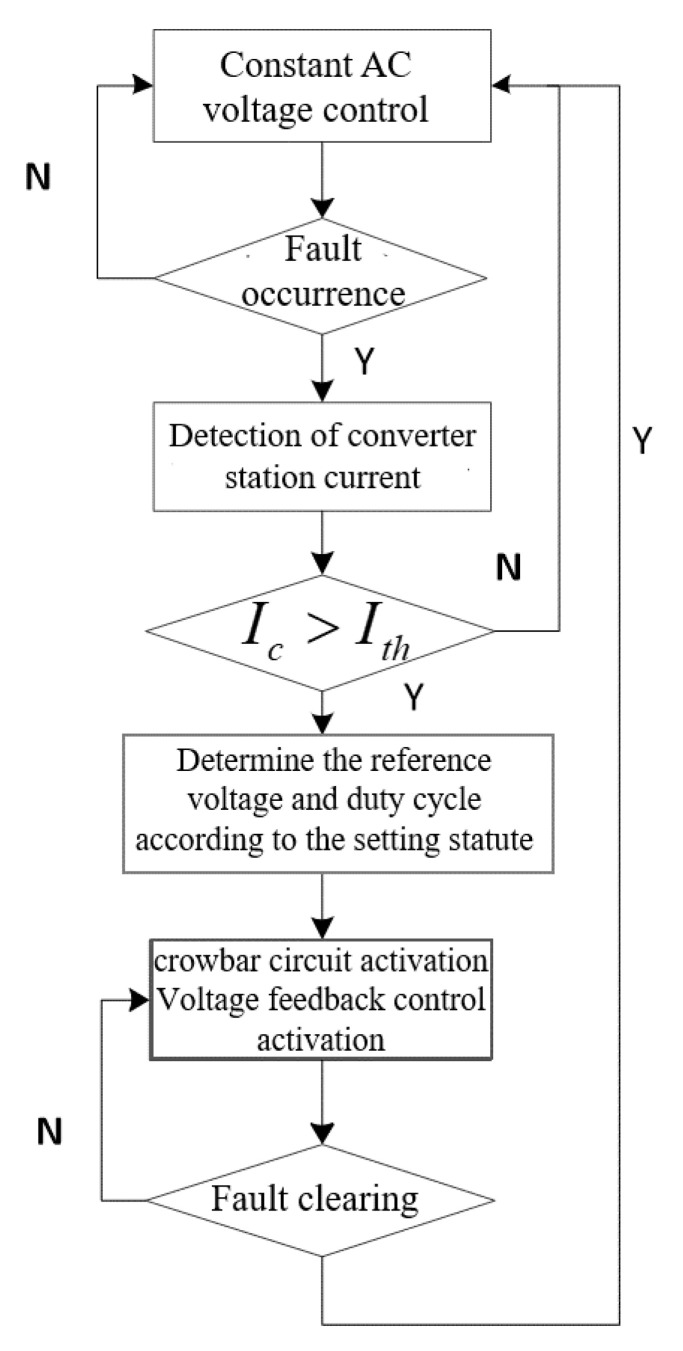
Procedure of coordinate FRT control strategy.

The control flow is shown in Figure 8:

## 5. Simulation Verification

In order to verify the performance of the coordinated fault ride-through control proposed in this paper, a model of a wind farm connected to the grid via a bipolar flexible DC transmission system was built at PSCAD/EMTDC. The wind farm model uses an equivalent model consisting of 33 DFIGs with a rated capacity of 1.5 MW. The simulation parameters are shown in Tables S1–S3 in Supplementary Materials.

### 5.1. Simulation 1

The simulation conditions are as follows: wind speed is 11 m/s, the initial value of the machine terminal voltage during fault ride-through is 0.9 p.u. The DC line fault occurs at 1.5 s, the IGBT is quickly blocked after the fault, the fault duration is 0.2 s, and the fault is removed at 1.7 s.

**Figure 9:**
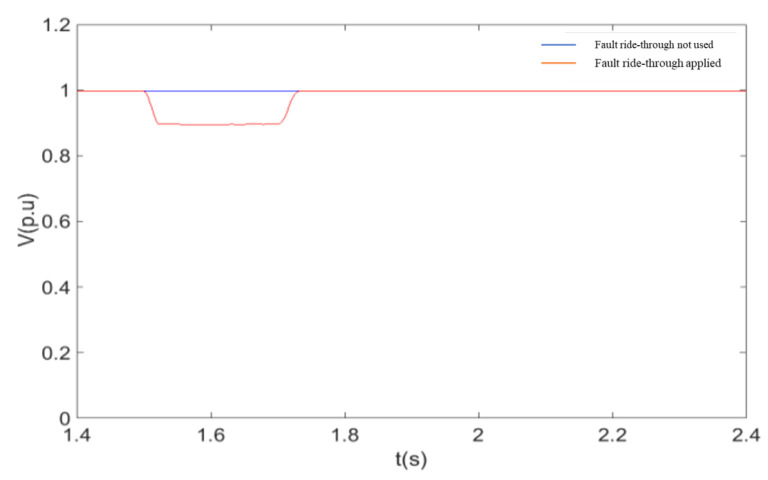
Soft straight non-fault pole active power.

**Figure 10:**
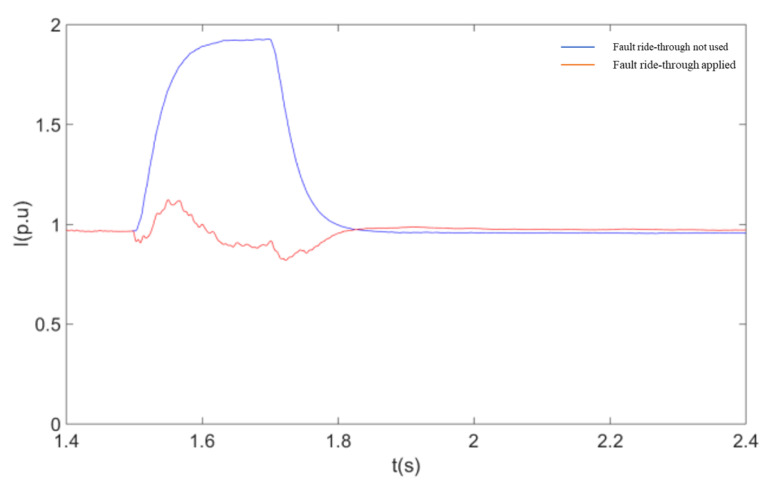
RMS Wind farm outlet voltage.

**Figure 11:**
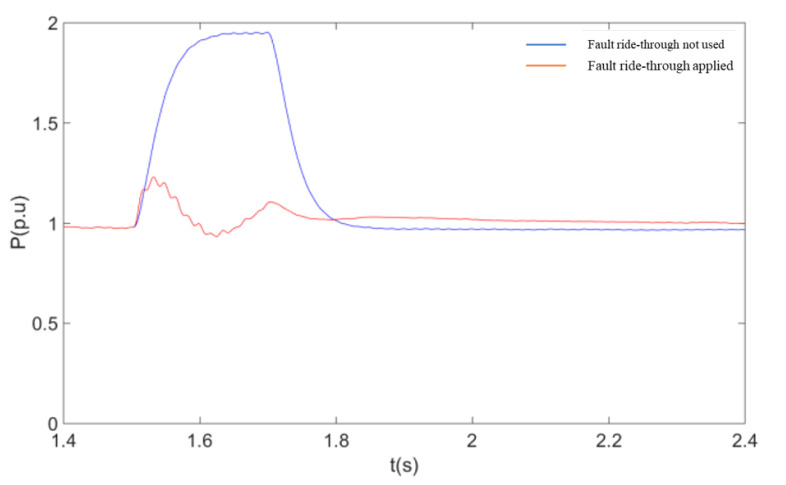
RMS value of flex-straight non-fault pole current.

As shown in Figure 9, when a single pole fault occurs in the flex-straight system, the wind farm outlet voltage remains almost constant during the fault because the WFVSC at the non-fault pole is still able to effectively control the magnitude and frequency of the wind farm outlet voltage. Figure 10 and Figure 11 give the effect of using a coordinated fault ride-through strategy with or without a coordinated fault ride-through strategy on the active power and the RMS value of the current at the non-faulted pole. From Figure 10 and Figure 11, after adopting the fault ride-through strategy, the rise of the non-fault pole current activates the coordinated fault ride-through strategy control and triggers the DFIG rotor-side crowbar circuit as the wind farm bus voltage decreases to the voltage reference value. With the rotor-side crowbar circuit engaged, the output power is reduced by about 45% compared to the non-fault ride-through reduction. Based on the above results, it is clear that the coordinated fault ride-through strategy can rapidly control the non-fault pole overcurrent caused by a single pole fault within the limit, thus realizing the fault ride-through of the whole system. At the same time, the WFVSC voltage signal and duty cycle can be adjusted to maintain a high delivery capacity at the non-faulted pole. After the end of the fault, both the flexible system and the wind turbine return to normal operation level, thus demonstrating that the coordinated fault ride-through strategy does not affect the operating status of the wind turbine and the DC system after the end of the fault.

The response of the system after a transient fault in a flexible mono-pole is shown in these diagrams:

### 5.2. Simulation 2

**Figure 12:**
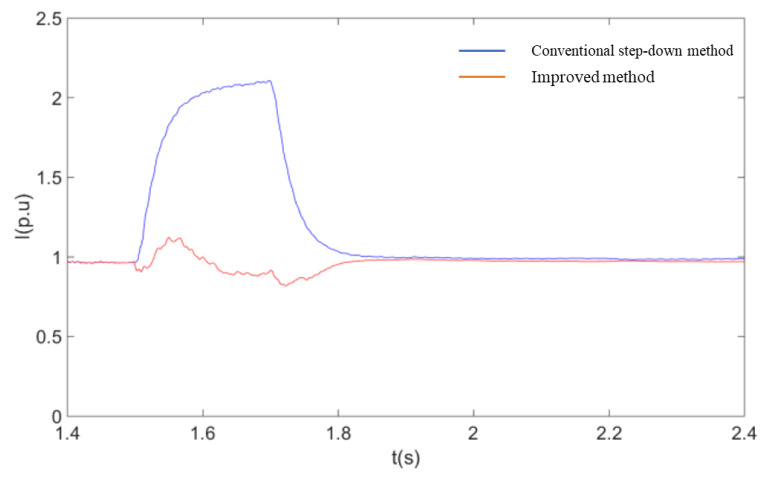
Soft straight non-fault pole current RMS.

**Figure 13:**
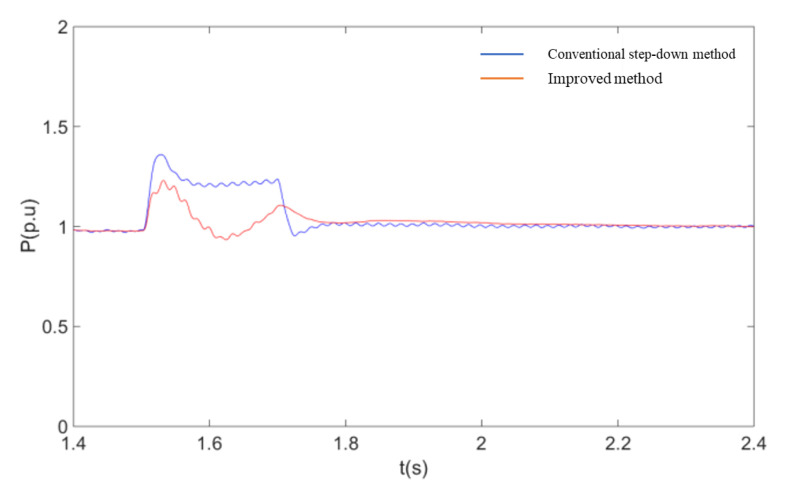
Flexible non-fault pole active power.

**Figure 14:**
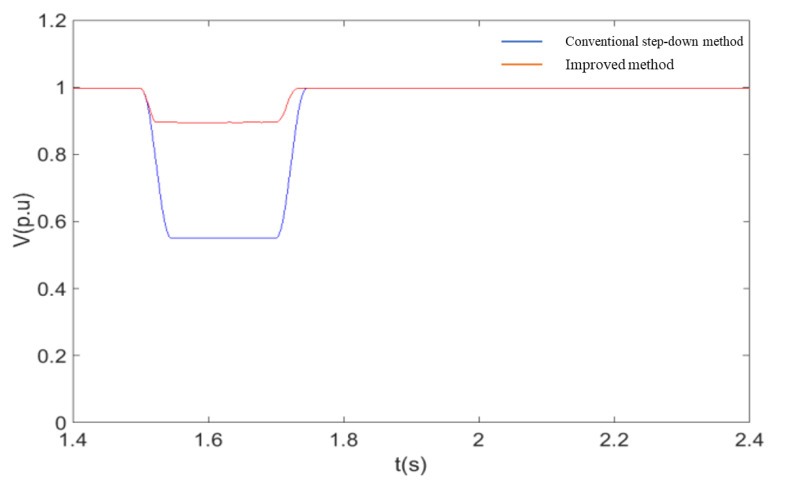
DFIG machine terminal voltage.

**Figure 15:**
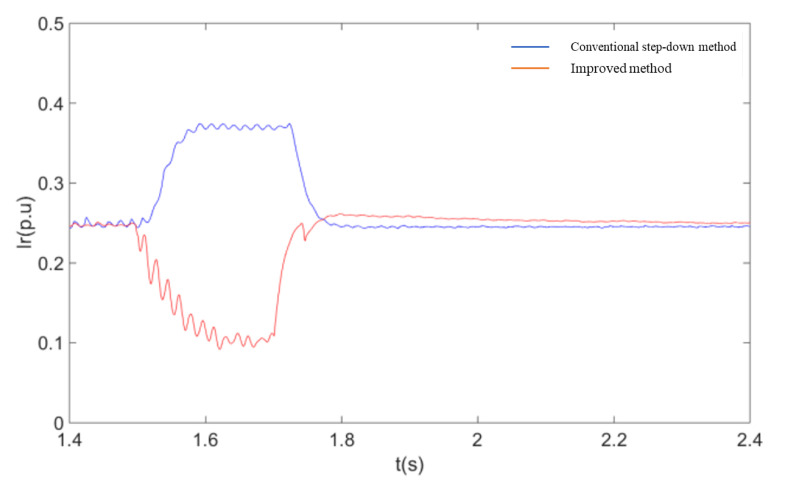
DFIG rotor current RMS.

In order to further verify the advantages of the coordinated control strategy described in this paper over the currently used method of accomplishing fault ride-through by simply reducing the voltage at the machine end, the two methods are compared through simulation experiments. The controller structure of the voltage reduction method uses the model given in the [14]. As can be seen from Figure 12, Figure 13, Figure 14 and Figure 15, when the conventional step-down method is used after a single-pole fault, the wind farm bus voltage drops to 58% of the rated voltage, and the active power flowing through the non-faulted pole is about 120% of the pre-faulted pole. Comparing the simulation results of Figure 12 with Figure 10, it can be seen that the current flowing through the non-faulted pole is not only not suppressed but also higher than the current value without the fault crossing when the conventional step-down method is used. At the same time, the use of the step-down method is equivalent to applying a large voltage drop at the stator side of the DFIG, and the current component induced by it causes a large increase in the rotor current, which brings a shock to the internal windings of the DFIG. Using the improved fault ride-through method described in this paper, the depth of the bus voltage drop is about 30% higher than that of the conventional step-down method for similar active power transfer at the non-faulted pole. The simulation results show that the method described in this paper has both a power suppression effect and a smaller voltage change compared with the conventional step-down method, so that the overcurrent at the non-faulted pole can be effectively limited.

A comparison of the response of the system under two different fault ride-through methods is shown in these following figures:

## 6. Conclusions

This paper presents a novel coordinated fault ride-through strategy for flexible DC transmission systems and wind farms, specifically targeting single-pole short-time faults in systems connected to the grid via bipolar flexible DC transmission in wind farms. The main contributions and findings of this study are outlined as follows:

(1) A comprehensive model of wind farms connected to the grid through a bipolar flexible DC transmission system is developed, capturing the system’s electrical characteristics.

(2) By analyzing the electrical behavior of the doubly fed induction generator (DFIG) after crowbar access, a coordinated fault ride-through strategy is proposed. This strategy combines the wind farm-side converter of the flexible DC transmission system with the crowbar circuit control of the wind turbine, leveraging the power output state of the wind farm cluster prior to the fault. Communication within the system is achieved using the wind farm bus voltage as the signaling mechanism. This approach allows fault ride-through of the flexible DC system without the need for additional communication links. Furthermore, it minimizes the impact on the wind turbine while maximizing the transmission capacity of the non-faulted pole.

(3) A joint operation curve for the wind farm voltage source converter (WFVSC) and wind turbine generators (WTGs) during fault ride-through is designed, taking into account the safety constraints of the DC system. During a single-pole fault in the DC system, the WFVSC adjusts the reference value of the AC bus voltage based on this operating curve, while the WTGs adapt the duty cycle of the crowbar circuit in response to changes in the bus voltage and input wind power. This ensures that the power output of the WTGs is suppressed, considering the variations in terminal voltage and internal impedance, thereby maintaining a non-current state in the non-faulted DC line during the fault.

(4) Simulation results confirm the effectiveness of the proposed coordinated fault ride-through strategy in reducing the non-faulted pole’s active power and current RMS. The strategy enables the fault ride-through of the entire system, without adversely affecting the operational state of the wind turbine and DC system after the fault is resolved. Additionally, the advantages of the coordinated control strategy over the traditional buck method for handling short-time faults in wind farms connected to the grid via flexible DC transmission with a single pole are demonstrated through comparative simulations. The results illustrate that the proposed method offers superior power suppression capabilities and smaller voltage variations compared to the traditional approach, effectively limiting overcurrent at the non-faulted pole.

(5) It is important to note that the proposed method is specifically applicable to single-pole short-time faults, owing to limitations in the crowbar’s heat-dissipation capabilities and other factors. Furthermore, this study focuses exclusively on the investigation of single-pole short-time faults in wind farms connected to the grid via flexible DC transmission.

Overall, this research contributes to the field of fault ride-through in wind farms and offers insights into the coordinated control strategy’s effectiveness in mitigating the impact of faults on flexible DC transmission systems. The findings provide a foundation for further advancements in fault ride-through strategies and contribute to the overall reliability and stability of wind farm integration with the grid.

## Data Availability

Data not available.

## Supplementary Materials

The following supporting information can be downloaded at: https://www.mdpi.com/article/10.3390/s23125760/s1.
