# Supplementary material for: Coordinate Fault Ride-Through Strategy for Connection of Offshore Wind Farms Using Voltage Source-Converter-Based High-Voltage Direct-Current Transmission under Single Polar Fault
Source: Sensors (Basel). 2023 Jun 20;23(12):5760. doi: 10.3390/s23125760 (PMC10302274; doi:10.3390/s23125760)
Supplement: Supplementary file 1 [file sensors-23-05760-s001.zip › sensors-2382557-supplementary.pdf]

Supplementary Materials

Section S1

In a VSC-HVDC system, DC voltage control is the control objective of the rectifier-side controller to balance the power transferred in the system. The variation of the DC side voltage is inherently related to the active power balance at both ends. If the DC current is constant, the DC side voltage variation will be proportional to the active power variation, then constant DC voltage control and constant active power control can be considered equivalent, so the DC voltage can be controlled by controlling the active current. Similarly, since the reactive power and reactive current of the system satisfy the positive correlation, the reactive power can be adjusted by changing the reactive current command value. The control block diagram is shown in Figure S1(a).

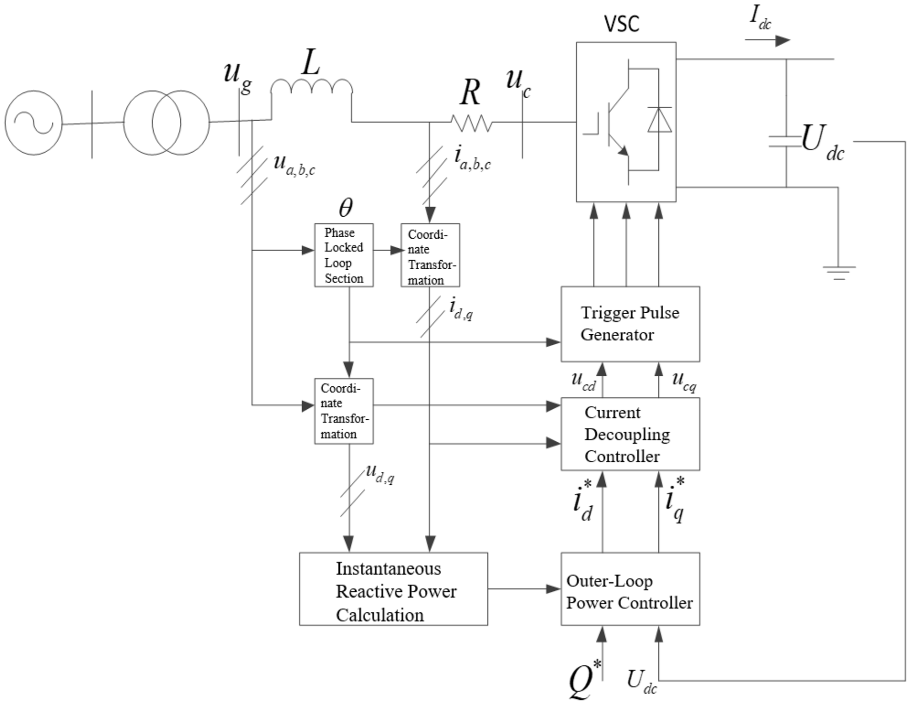

Figure S1. (a) GSVSC control strategy.

The VSC-HVDC converter station connected to the DFIG must establish a stable AC voltage. The error between the set wind farm end voltage reference value V_{wfref} and the actual wind farm end voltage V_{wf} is controlled by PI, and the frequency of the three-phase sine wave generator is set to f_{ref}, and the three-phase sine wave generator is designed to be three-phase symmetric , which means that the target value of the three-phase voltage fundamental component on the commutation bridge side can be obtained. Then the sine pulse width modulation (SPWM) method is used to control the on and off of the IGBT. The control block diagram is shown in Figure S1(b).

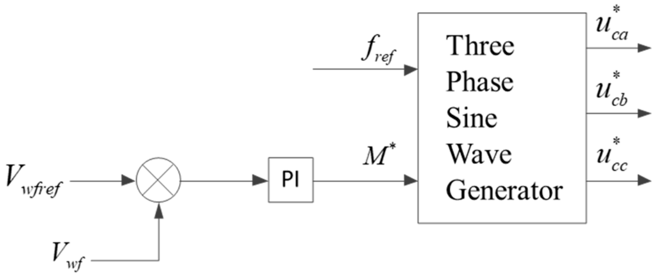

Figure S1. (b) WFVSC control strategy.

The active power control of the DFIG is achieved through a combination of maximum wind power tracking (MPPT) control and a pitch angle control system. The control system obtains the reference value of active power by the MPPT algorithm and provides it to the DFIG rotor-side converter RSC, which eliminates the coupling between active and reactive power by means of the stator chain directional vector control technique, i.e., the active power of the doubly-fed generator can be controlled by controlling the q-axis component of the rotor current, and the d-axis component of the rotor current can be controlled by controlling the reactive power of the doubly-fed generator. reactive power by controlling the d-axis component of the rotor current. Thus, the output active power and reactive power can be controlled independently and the DFIG can be operated at variable speed and constant frequency. The control diagram of the rotor-side PWM converter is shown in Fig. S2(a).

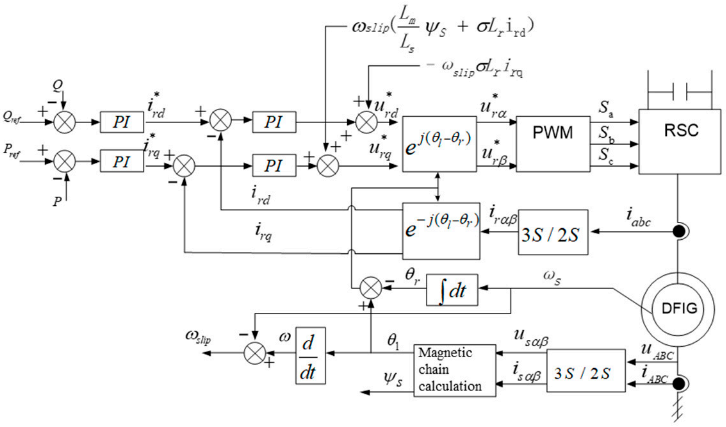

Figure S2. (a) RSC control strategy.

The task of the grid-side converter is to keep the DC bus voltage stable, the input current sinusoidal and to control the input power factor. The stability of DC voltage is closely related to the active power balance between AC side and DC side. Under the condition of constant grid voltage, the control of AC side active power is to control the active component of input current; the control of input power factor is actually to control the reactive component of input current; the sinusoid of input current waveform is mainly related to the effectiveness of current control and modulation mode. Therefore, the control system of the network-side converter is divided into two parts: voltage outer-loop control and current inner-loop control. The control diagram of the grid-side PWM converter is shown in Figure S2(b).

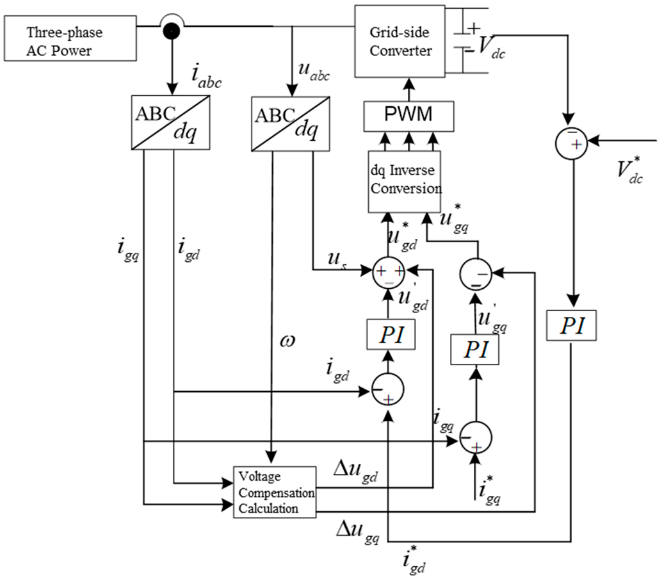

Figure S2. (b) GSC control strategy.

Section S2

The IGBT type Crowbar circuit consists of an uncontrolled rectifier bridge and an IGBT fully controlled device. Each bridge arm is connected in series with two diodes, while the DC side consists of an IGBT and an absorber resistor. The generator rotor current can be freely regulated by controlling the trigger signal of the IGBT, thus suppressing the generator rotor overcurrent and allowing the unit to be controlled by reintroducing the converter when the grid voltage is restored.

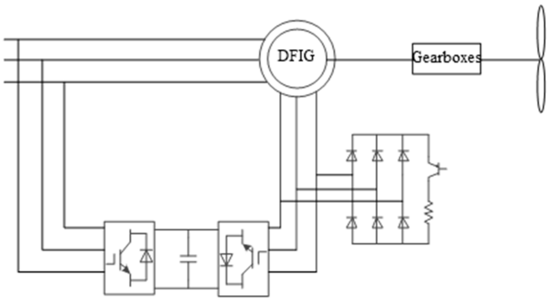

Figure S3. IGBT type Crowbar circuit.

Section S3

When the Crowbar circuit is connected, the voltage equation of DFIG is as follows:

$$\begin{cases} u_{sd} = R_s i_{sd} + p\psi_{sd} - \omega\psi_{sq} \\ u_{sq} = R_s i_{sq} + p\psi_{sq} + \omega\psi_{sd} \\ u_{rd} = R_r i_{rd} + p\psi_{rd} - s\omega\psi_{rq} \\ u_{rq} = R_r i_{rq} + p\psi_{rq} - s\omega\psi_{rd} \end{cases} \quad \text{(S1)}$$

The equation of the magnetic chain is as follows:

$$\begin{cases}\psi_{sd}=L_s i_{sd}+L_m i_{rd}\\ \psi_{sq}=L_s i_{sq}+L_m i_{rq}\\ \psi_{rd}=L_m i_{sd}+L_r i_{rd}\\ \psi_{rq}=L_m i_{sq}+L_r i_{rq}\end{cases} \tag{S2}$$

The electromagnetic power expression is as follows:

P

(S3)

where u_{sd}, u_{sq}, u_{rd}, u_{rq} are the d, q-axis components of the stator-rotor phase voltage, respectively; i_{sd}, i_{sq}, i_{rd}, i_{rq} are the d, q-axis components of the stator-rotor phase current, respectively; ψ_{sd}, ψ_{sq}, ψ_{rd} ψ_{rq} are the d, q-axis components of the stator-rotor magnetic chain, respectively; L_m is the mutual inductance between the stator-rotor coaxial equivalent windings in the dq coordinate system; L_s is the stator equivalent two-phase winding self-inductance in dq coordinate system; L_r is the rotor equivalent two-phase winding self-inductance in dq coordinate system; ω is the synchronous speed of DFIG.

Substituting equation (S2) into equation (S1) and neglecting the transient process of the stator-rotor magnetic chain, i.e. $p\psi_{sd}=0$, $p\psi_{sq}=0$. Substituting the calculated stator-rotor current dq-axis component into equation (S3), the expression of electromagnetic power is obtained as:

$$P_e=\frac{s\omega_s^2\sigma L_m L_s L_r[L_r^2 R_s+L_m^2 R_r+(s^2\omega_s^2)\cdot(L_r^2 R_s+L_s L_r R_r)]u_{sd}^2}{(R_r L_s+R_s L_r)^2+(\omega_s\sigma L_r L_s)^2} \tag{S4}$$

where $\sigma=1-L_m^2/L_sL_r$

Section S4

Table S1. The parameter of wind turbine.

Wind turbine parameters	Parameter value/unit
Rated power	1.5MW
Blade radius	40.0m
Optimum blade tip speed ratio	8.36
Optimal wind energy utilization coefficient	0.42
Air density	1.225kg/m^3

Table S2. The parameter of DFIG.

DFIG parameters	Parameter value/unit
Stator Voltage	0.69kV
Stator resistance	0.0054 p.u.
Stator leakage inductance	0.10 p.u.
Stator-rotor mutual inductance	4.5 p.u.
Rated power	1.5MW
Rotor resistance	0.00607 p.u.
Rotor leakage inductance	0.11 p.u.
Inertia time constant	0.85
Energy discharging resistance	1.51p.u.

Table S3. The parameter of VSC-HVDC.

VSC-HVDC parameters	Parameter value/unit
Flat wave reactors	5 mH
Converter resistance value	0.2 Ω
Coupling transformer rated voltage	36.5/31
SPWM carrier frequency	3000Hz
AC side rated voltage	35kV
DC side rated voltage	±30 kV
DC side rated current	250A
Converter capacitance value	2500 μF
DC line resistance value	0.01 Ω
DC line inductance value	1 mH
